# Cooperative Effect of Chemical and Physical Processes for Flame Retardant Additives in Recycled ABS

**DOI:** 10.3390/polym15112431

**Published:** 2023-05-24

**Authors:** Alicia Rodriguez, Manuel Herrero, Maria Asensio, Mercedes Santiago-Calvo, Julia Guerrero, Esteban Cañibano, Maria Teresa Fernández, Karina Nuñez

**Affiliations:** 1 Foundation for Research and Development in Transport and Energy (CIDAUT), Parque Tecnológico de Boecillo, 47051 Valladolid, Spain; 2 Department of Architectural Construction, Geotechnical Engineering, Continuum Mechanics and Structure Theory, School of Industrial Engineering, University of Valladolid, Paseo del Cauce 59, 47011 Valladolid, Spain

**Keywords:** acrylonitrile-butadiene-styrene, recycled, non-halogenated flame retardants, mechanical properties

## Abstract

In the present work, the effectiveness of four non-halogenated flame retardants (FR) (aluminium trihydroxide (ATH), magnesium hydroxide (MDH), Sepiolite (SEP) and a mix of metallic oxides and hydroxides (PAVAL)) in blends with recycled acrylonitrile-butadiene-styrene (rABS) was studied in order to develop a more environmentally friendly flame-retardant composite alternative. The mechanical and thermo-mechanical properties of the obtained composites as well as their flame-retardant mechanism were evaluated by UL-94 and cone calorimetric tests. As expected, these particles modified the mechanical performance of the rABS, increasing its stiffness at the expense of reducing its toughness and impact behavior. Regarding the fire behavior, the experimentation showed that there is an important synergy between the chemical mechanism provided by MDH (decomposition into oxides and water) and the physical mechanism provided by SEP (oxygen barrier), which means that mixed composites (rABS/MDH/SEP) can be obtained with a flame behavior superior to that of the composites studied with only one type of FR. In order to find a balance between mechanical properties, composites with different amounts of SEP and MDH were evaluated. The results showed that composites with the composition rABS/MDH/SEP: 70/15/15 wt.% increase the time to ignition (TTI) by 75% and the resulting mass after ignition by more than 600%. Furthermore, they decrease the heat release rate (HRR) by 62.9%, the total smoke production (TSP) by 19.04% and the total heat release rate (THHR) by 13.77% compared to unadditivated rABS; without compromising the mechanical behavior of the original material. These results are promising and potentially represent a greener alternative for the manufacture of flame-retardant composites.

## 1. Introduction

The increasing use of plastic materials to replace metal parts in sectors where high fire protection requirements are needed, such as automotive, aeronautics, construction, and electrical-electronic, has increased the demand for high-performance flame retardant (FR) plastics.

The incorporation of FR additives into plastics has been used as a method to provide fire protection properties to materials [1]. These flame retardant additives act by delaying the release of toxic gases during fire by increasing the burning time of the polymer matrix. Therefore, FR additives are essentially designed to save lives, and for this reason the worldwide consumption of FR additives was over 2.49 Mt in 2015 and the market size is expected to exceed 4.0 million tons by 2025 [2].

Different types of additives are used to provide flame-retardant functionality to plastic materials, among which mainly two types of FRs are used: halogenated and non-halogenated. For years, halogenated FRs (HFRs) containing bromine or chlorine have been the most widely used in plastic applications due to the higher efficiency. The mechanism of the HFRs is the chemical transformation of halogen into radicals to capture free radicals during the combustion process, which hinder the combustion of the polymer matrix. However, HFRs have disadvantages such as the release of large amount of toxic gases and smoke, which promotes environmental pollution [3,4]. Thus, the use of these FRs is being questioned as environmental concerns have led to several countries to establish regulations to limit their use [5].

Another more environmentally friendly solution used to provide plastics with fire resistance functionality is the use of halogen-free FRs or non-halogenated FRs additives [6]. The flame-retardant mechanism of these FRs additives is based on the cooling effect of the solid phase by releasing non-flammable water molecules as well as promoting the formation of a protective layer. The most commonly FRs used are metal hydroxides (aluminium and magnesium hydroxides). However, the low efficiency of metal hydroxides usually requires a very high loading of at least ≥50 wt.%, which often negatively affects the mechanical and processing properties of the polymer composites [7]. These drawbacks have promoted research into environmental-friendly FR with good fire performance, but which also maintain plastic behavior for processing into final products. The effect of non-halogenated FRs has been thoroughly investigated by focusing only on virgin polymers. One of the most demanded polymers for fire protection applications in industry is ABS, especially for electronics and automotive components [8]. There are numerous studies related to ABS with non-halogenated FR and there are even commercial references for obtaining engine casings, electronic components, etc. [9,10]. Several studies have been carried out on recycled ABS in terms of improving thermo-mechanical properties by using nanoclays or other virgin polymers [11,12]. However, studies focusing on improving the flame retardant properties of recycled polymers are scarce [13,14,15,16,17]. This shows that the development of flame retardant recycled ABS materials is a niche with many opportunities for future development.

Based on this, the present research aims to study for the first time the effect of environmentally friendly FR additives in a recycled acrylonitrile-butadienestyrene (rABS) material. This work evaluates the effectiveness of different FRs,—such as aluminium trihydroxide (ATH), magnesium hydroxide (MDH), Sepiolite (SEP) and PAVAL-, incorporated into rABS by extrusion. The effects of the addition in terms of mechanical and fire behavior were studied, as well as their synergistic effect. Therefore, this research aims to remove hazardous FRs in future polymer streams and, consequently, in future polymer waste streams.

## 2. Experimental Section

### 2.1. Materials

The base polymeric matrix used for the different formulations was a white rABS provided by COOLREC from the CREATOR Project [18]. The material was subjected to a purification process before being used as a raw material for the preparation of the characterization samples. The additives selected for the preparation of the FR composites were ATH (Martinal, ON313S), MDH (KMT Industrial, XK-325/92), SEP nanoclay (Tolsa, Pangel S9) and PAVAL. The latter material was kindly provided by BEFESA.

The FR additives were selected according to their potential use as a FR, their commercial availability and/or cost, as shown below:

- ATH is the most common non-halogenated FR but has low commercial availability and a higher cost.
- MDH is also used as a FR additive but is more expensive than ATH.
- PAVAL is an aluminium industrial waste stream that can be used as alternative to ATH at lower cost. This material mainly consists of alumina and other oxides, aluminium nitride and carbide, and is usually disposed in landfills as a non-hazardous material, sold to cement producers or used in calcium aluminate production [19,20].
- Sepiolite (SEP) is based on low-cost phyllosilicates that have the ability to produce synergies when mixed with ATH or MDH [21].

### 2.2. FR Composites Preparation

The rABS matrix and additives were dried at 80 °C for at least 24 h before processing. After that, the composites were produced by extrusion process using a co-rotating twin-screw extruder, model Leistritz 27GL (L/D = 36). In all references, the rABS were dosed from the main hopper, while the FR additives were added through the side port (chamber 5 from the die) of the extruder by gravimetric dosing.

**Figure 1:**
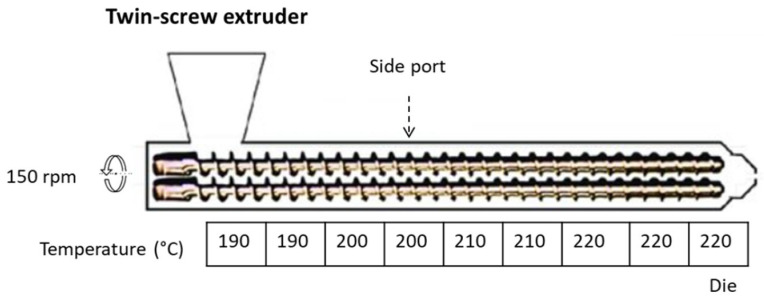
Processing conditions of the extrusion samples.

The first set of FR composite samples and the pure rABS were extruded at a temperature profile ranging from 190 to 220 °C (see Figure 1) and at 150 rpm to ensure good dispersion of the additives without polymer matrix degradation.

The total amount of additives in the samples was kept constant throughout the experiments (15 wt.%). A list of the composition of the samples is listed in Table 1. References [2,3,4,5] were prepared using only one FR to study the effect of each additive, while the references [6,7,8,9,10,11] were prepared by mixing two different FR additives to study potential synergies between them.

### 2.3. Characterization

#### 2.3.1. Characterization of FR Additives

A complete characterization of the selected FR additives was carried out. Tests included: density, particle size, water release capacity and temperature.

Density was measured with a liquid pycnometer according to ISO 1183 Method B. Particle size analysis was conducted using a FESEM Hitachi H-7000 scanning electron microscopy (SEM), taking three SEM images of each FR additive and measuring the size of at least fifty particles to report the average values. Finally, water release capacity and release temperature were determined by TGA. TGA thermograms were performed in nitrogen atmosphere with a heating of 25 °C/min (25–900 °C) using a Mettler Toledo equipment, model TGA/SDTA851e.

#### 2.3.2. Characterization of FR Composites

- Thermogravimetric analysis (TGA)

The final amount of FR added in each composite sample was calculated by TGA. TGA curves were obtained from the pelletized extruded samples in nitrogen atmosphere at a heating rate of 25 °C/min (25–1100 °C) using a Mettler Toledo equipment, model TGA/SDTA851e.

- Tensile Test

Young’s modulus and tensile strength were measured at room temperature using an Instron Model 5500R60025 at a speed of 1 mm min^−1^ and 50 mm min^−1^, respectively, and according to ISO 527-1. The tensile test specimens were injected with dimensions type 1A on a Krauss Maffei KM 200 injection molding machine. The temperature profile of the cylinders was 200 to 220 °C and the mold temperature was 60 °C. For each reference, five specimens were tested and the mean values were given.

- Heat Distortion Temperature (HDT)

Service temperature of the FR composites was measured on a CEAST HDT3-VICAT P/N 6911/000, using a load of 1.8 MPa, according to ISO 75. The specimens were injected following the same procedure as for the tensile tests and were mechanized with dimensions of 80 mm × 10 mm × 4 mm. For each reference, three specimens were tested and the mean values were reported.

- Charpy Impact

Charpy V-notch tests were carried out on a Resil 6957 impact pendulum at room temperature according to ISO 179. The specimens were injected following the same procedure as for the tensile tests and mechanized to dimensions of 80 mm × 10 mm × 4 mm. For each reference, eight specimens were tested and the mean values were calculated.

- Burning Test (UL-94HB)

UL 94 is a flammability standard for plastics released by Underwriters Laboratories (Northbrook, IL, USA). This standard classifies plastics according to how they burn in various orientations and thicknesses, from the least flame-retardant to most flame-retardant, into six different classifications (HB, V-2, V-1, 5VB and 5VA). In the case of the samples under study, the horizontal burning test was carried out following UL-94HB, which is technically equivalent to ASTM D635 (burning rate and/or extent and burning time of plastics in horizontal position). The specimens were injected following the same procedure as for the tensile tests and were mechanized with dimensions 80 mm × 10 mm × 3 mm. For each reference, five test specimens were tested and mean values were reported.

- Cone Calorimeter Tests

Cone calorimeter tests were performed with a cone calorimeter apparatus (FTT, Fire Testing Technology, East Grinstead, UK) under an external heat flux of 50 kW/m^2^ according to ISO 5660-1. The specimens were injected with dimensions of 100 mm × 100 mm × 4 mm. For each reference, three specimens were tested and their average data were reported.

## 3. Results and Discussion

### 3.1. Characterization of FR Additives

A full characterisation of the selected FR additives was carried out where density, particle size, water release capacity and temperature were analyzed. A full description of this characterisation is given in Section 2.3.

**Figure 2:**
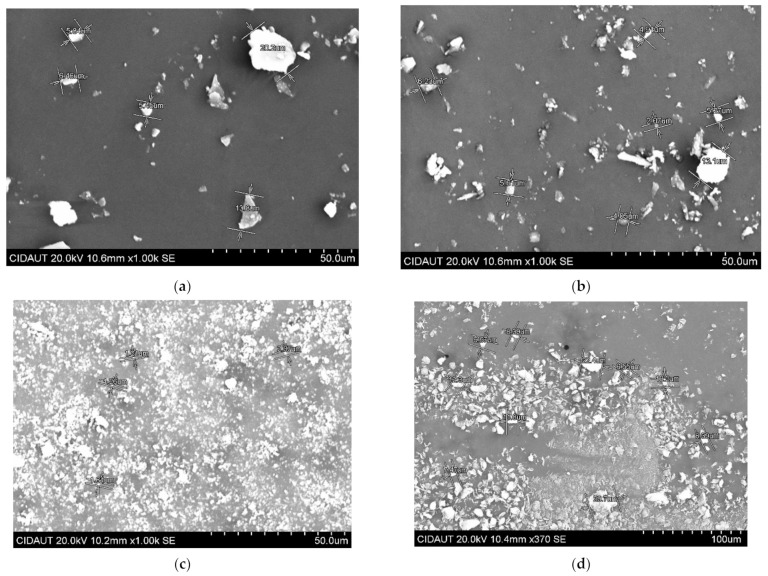
SEM images of the different FR additives. (**a**) ATH. (**b**) MDH. (**c**) PAVAL. (**d**) SEP.

The amount, size and shape of the additives have an impact on the dispersion in the polymers, which influences the final properties of the material. For example, to improve the flame behavior, polymers need large amounts of FR microadditives, but this will be detrimental to the mechanical properties of the final composite [22]. However, if the size of the additives is small, as in the case of FR nanoadditives, a reduction in their quantity can be achieved with respect to larger additives, increasing the surface area per unit volume and improving the interactions between the additives and the polymer matrix avoiding a loss of mechanical properties [23]. Therefore, in order to know the particle size of the different FR additives, SEM images were taken (Figure 2). The average size of each additive is shown in Table 2.

It can be observed that the metallic hydroxides (ATH and MDH) have a similar particles size (Figure 2a,b), while PAVAL and SEP present an order of magnitude smaller size (Figure 2c,d). At this point, it is important to mention that SEP is a needle-shaped nanoclay and that these measurements were performed on agglomerates in their native state, due to their high specific surface area. However, our previous studies showed that SEP dispersed perfectly under the extrusion conditions used [24,25]. In all cases the additives presented small sizes (≤20 µm) which make them suitable to be used as additives to obtain composites with good dispersion.

To improve flame retardancy with non-halogenated additives, it has already been mentioned that large amounts of FR additives are needed. The addition of these additives has a large influence on the final density of the composite materials, which can limit their use in specific applications. Table 2 shows the density of the different FRs. According to the data, hydroxides (ATH and MDH) have higher density than PAVAL and SEP, leading to higher density formulations for similar loadings within the polymer matrix. This means that formulations containing ATH and MDH will experience an increase in the weight of the final component.

**Figure 3:**
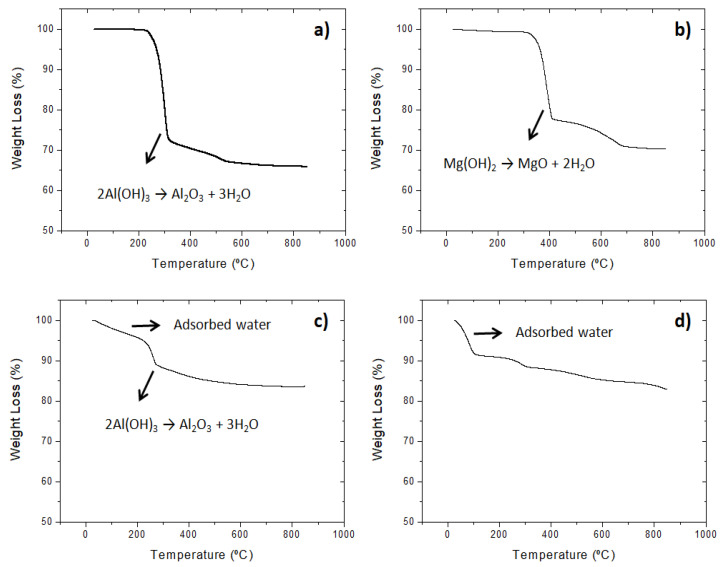
TGA analysis of the different FR additives: ATH (**a**), MDH (**b**), PAVAL (**c**) and SEP (**d**).

It is well known that the mechanism of metal hydroxides against fire is based on the release of water during their decomposition [26,27]. Therefore, it is important to investigate two key parameters: water generation and release temperature. To obtain these data, a TGA analysis of the different additives was performed (Figure 3) and the results can also be seen in Table 2.

Regarding water release, the results showed that the FRs based on oxides and hydroxides (MDH, ATH and PAVAL) have the highest capacity to release water during their calcinations, while the SEP water realisation recorded in the table corresponds to the evaporation of surface water at 100 °C as shown in the TGA curve (Figure 3d).

According to the results, the amount of water released follows the trend: ATH, MDH and PAVAL (Figure 3a–c). This result is closely related to the molecular structure and composition of each additive. ATH releases three water molecules during its conversion into alumina, while MDH releases only two water molecules during its decomposition into magnesium oxide. PAVAL showed a significantly lower water release due to the fact that only about 40 wt.% of its composition is aluminium trihydroxide.

The water release temperature showed the following trend: MDH ˃ ATH ˃ PAVAL. In all cases, the release temperatures were higher than the processing temperature of the rABS composites, which ensures that the additives do not decompose during the extrusion and injection moulding. Nevertheless, it has been observed that in other polymeric matrices with a higher melting temperature, the use of ATH and PAVAL as FR additives can lead to decomposition during the melting compounding process. In the case of SEP, as mentioned above, water release is related to the adsorption of water molecules due to the high density of hydroxyl groups on its surface. However, it is important to note that this surface water evaporates during the drying process before extrusion. Therefore, the adsorbed water does not affect the ABS processing process.

**Figure 4:**
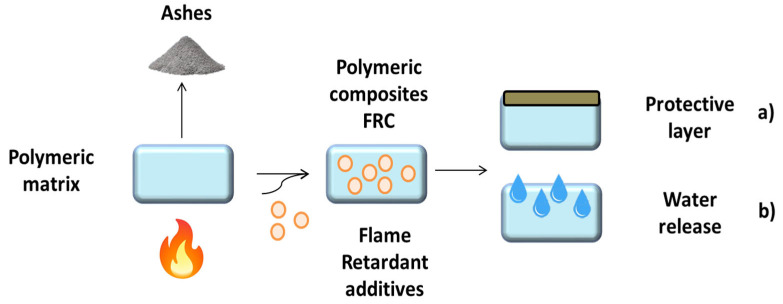
Proposed flame retardant mechanism for different additives: SEP (**a**) and ATH, MDH, PAVAL (**b**).

Based on the TGA analysis, it is possible to propose two mechanisms for the investigated FR additives (Figure 4). On the one hand, ATH, MDH and PAVAL have a mechanism based on the decomposition of the hydroxides into their corresponding oxides. This reaction contributes to flame retardancy in three ways [28,29]: (1) the decomposition of the FR which is an endothermic process, cooling the temperature of the polymer matrix; (2) the release of water vapour which dilutes the combustible gases and forms a protective gas layer; (3) the formation of oxides during combustion, which acts as an insulating ceramic coating against fire. On the other hand, SEP nanoclays show a different mechanism acting as a pure barrier against flame propagation. When the nanoclays are well dispersed, they hinder the macromolecular mobility that affects the degradation pathway during combustion. Moreover, during flame propagation through the composite, the nanoclays, which have a high decomposition temperature (>1000 °C), are converted into an insulating char structure that dissipates the incident heat [30,31].

### 3.2. Characterization of FR Composites with ATH, MDH, PAVAL and SEP

#### 3.2.1. Mechanical Properties

**Figure 5:**
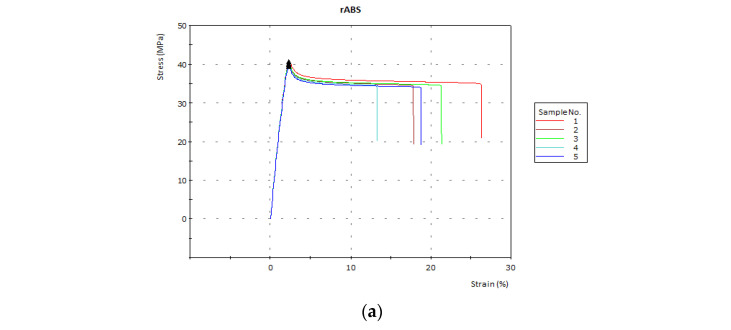

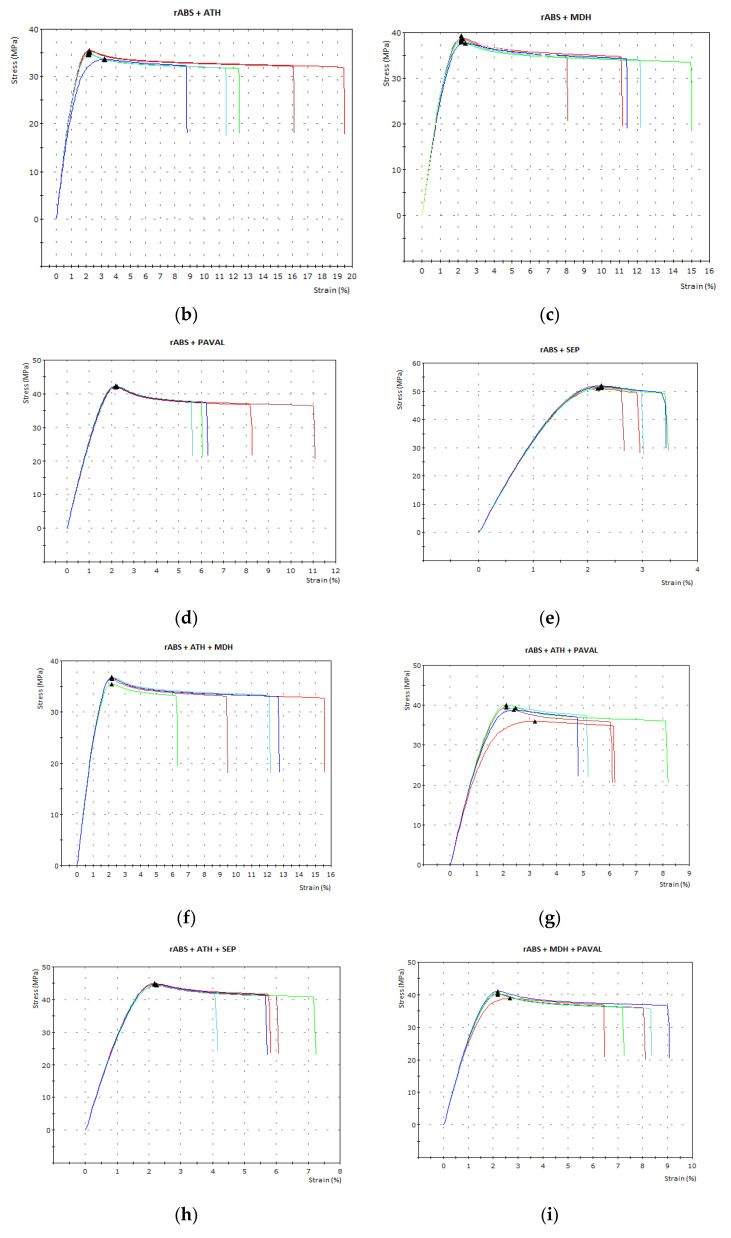

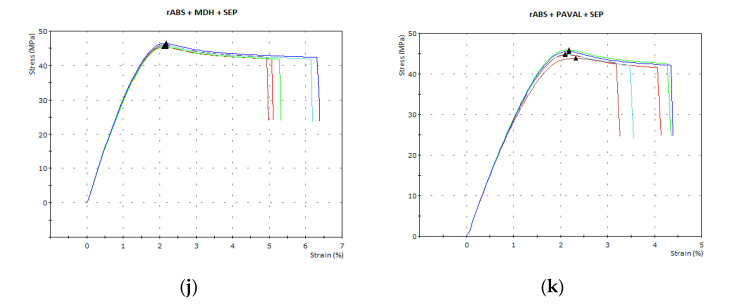
Tensile tests graphs of the different FR composites (the black triangle shows the strength values (σ) of each sample): (**a**) rABS, (**b**) rABS + ATH, (**c**) rABS + MDH, (**d**) rABS + PAVAL, (**e**) rABS + SEP, (**f**) rABS + ATH + MDH, (**g**) rABS+ATH + PAVAL, (**h**) rABS + ATH + SEP, (**i**) rABS + MDH + PAVAL, (**j**) rABS + MDH + SEP, and (**k**) rABS + PAVAL + SEP.

**Figure 6:**
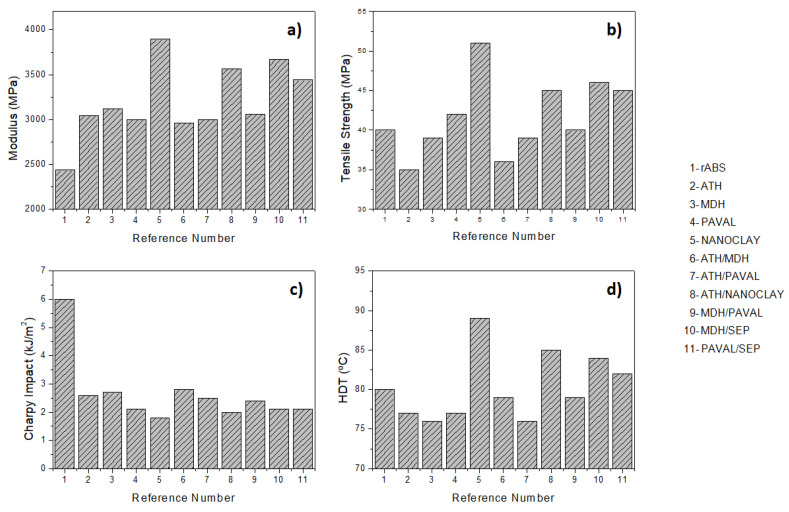
Thermo-mechanical properties of FR composites: modulus (**a**), tensile strength (**b**), Charpy impact (**c**) and HDT (**d**).

After injection molding all references were subjected to thermo-mechanical characterization based on tensile, Charpy impact and HDT tests. The result of this characterization program provides useful information about the behavior of the composite materials when used on a large scale in the final application. These results are shown in Table 3, Figure 5 and Figure 6.

Different trends were observed in the tensile tests as can be seen in Figure 5.

In this case, the addition of ATH, MDH, PAVAL and their blends leads to similar or lower strength values (**σ**) compared to rABS. This phenomenon is due to the fact that these additives are not able to contribute to stress transfer with the polymer matrix during tensile tests [32]. On the contrary, the references containing SEP presented tensile values higher than those of the starting polymer. As in the case of the modulus, SEP demonstrated its ability to reinforce different polymer matrices [33,34]. The highest value was observed in Reference [5], to which 15 wt.% SEP had been added. This reference showed a value of 51 MPa for tensile strength, which represents an increase of 22% compared to rABS. The other references based on a blend containing SEP (7.5 wt.%) also showed significantly higher values than the starting polymer matrix.

Regardless of the additive used, the addition of FR leads a decrease in the elongation at break values (**ε**). It is well known that the addition of inorganic particles within polymer matrices causes a reversal from ductility to brittleness, causing a loss of plastic deformation during tensile tests [35,36].

Focusing on the Young’s modulus results, the first important observation is the increase of the values with the addition of FR additives (Figure 6a). The references based on ATH, MDH, PAVAL and their blends result in composites with modulus values centered on 3000 MPa, which represents an increase of 20% compared to the starting rABS. The best modulus values were obtained for the references containing SEP. The SEP-based material (Reference [5]) was the composites with the highest modulus value, representing an increase of 37%, while the references based on a mixture containing SEP (References [8,10,11]) presented values centered on 3500 MPa (≈30%). The increase in modulus values is due to the addition of the inorganic particles, which provides stiffness to the starting rABS. The improvement of the modulus results in the composites containing SEP was due to their nano-sized and fibrillary structure, which favoured the interaction between the SEP and the polymer matrix increasing the stiffness of the different polymeric matrices.

Another important thermo-mechanical property evaluated was the ability of FR composites to absorb energy during impacts. As mentioned above for the elongation at break values, the addition of FR additives leads to a more brittle behavior of the materials and, consequently, to a decrease in the energy absorption capacity. For this reason, nanocomposites generally do not exceed 10 wt.% [37]. Regardless of the additive used, the Charpy values decrease from 6.25 kJ/m^2^ for rABS to ≈2.5 kJ/m^2^ for all composites loaded at 15 wt.%, which is a 60% drop compared to the starting material (Figure 6c). At this point, it is important to mention that the ABS used as polymeric matrix in this study is a recycled fraction, which presents a lower initial value compared to pure ABS (≈20 kJ/m^2^).

HDT tests were used to evaluate the service temperature of the composite materials (Figure 6d). The HDT test is defined as the temperature at which the deflection of a standard specimen reaches 0.25 mm under a maximum applied stress of 1.82 MPa. The results showed that the formulation based on ATH, MDH and PAVAL led to a slight decrease in the service temperature (≤4 °C). However, the references containing SEP increased HDT values. Thus, references containing 7.5 wt.% SEP showed slightly higher values (from 2 °C to 5 °C), while Reference [5] showed the highest HDT value, which increased of 9 °C compared to rABS. This trend, which has also been observed in tensile tests, reinforces the argument that only SEP is able to improve stress transfer during mechanical testing.

#### 3.2.2. Fire Properties

The fire behavior of the obtained composites was evaluated using the UL-94 HB test to study the tendency of the materials to spread and extinguish the flame when the materials are ignited by applying a flame for 30 s.

Table 4 shows the burning results: burning with/without dripping, linear burning rate and flammability rating. One of the main observations was that during the tests none of the references self-extinguished. However, in all cases the addition of FR additives led to a significant decrease in the burning rate. In the case of mono-additivated references, only the sample containing SEP [5] passed the UL-94 HB test, as it showed a burning rate of less than 40 mm/min. The rest of the mono-additivated samples [2,3,4] showed burning rates slightly higher than 40 mm/min and were therefore not classified by the UL-94 HB test. All the mono-additivated samples that were oriented horizontally showed dripping during flame application. In the case of bi-component FR formulations, only [10] (MDH + SEP) and [11] (PAVAL + SEP) passed the HB classification. Focusing on the overall improvement from the baseline rABS, [10] showed an improvement of more than 40%, which is the largest improvement observed. Thus, Reference [10] showed a synergistic effect between Sepiolite and MDH when used together. Furthermore, it is important to note that among all FR composites, [8,10,11], based on a mixture of SEP with another additive, were the only ones of which no dripping occurred during combustion. In addition, a reduction of dripping was observed in the samples containing FR compared to the reference material (rABS). In conclusion, among all the materials classified by the UL-94 HB tests, the best result was reached with the sample containing MDH and SEP [10], as this material had the lowest burning speed (V32) and no dripping occurred during its combustion.

### 3.3. MDH/SEP Composites

According to previous results, the use of SEP led to composites with improved mechanical properties and its combination with MDH allowed for composites with better fire performance (no dripping and low burning rates) to be obtained. Therefore, the effects on the mechanical and fire properties of the final composites with different amounts of FR were systematically studied. Table 5 shows the comparison between the programed wt% of FR and the final amounts measured by TGA. It can be seen that the FR content in the samples was close to the desired value.

#### 3.3.1. Mechanical Properties

**Figure 7:**
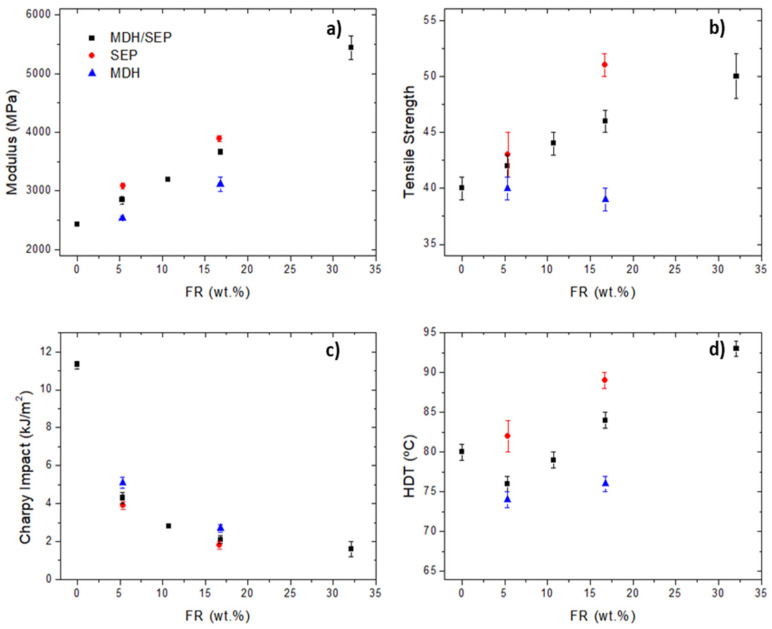
Thermo-mechanical properties of the composites as a function of FR additives and quantity: modulus (**a**), tensile strength (**b**), Charpy impact (**c**) and HDT (**d**).

The results of the thermo-mechanical tests are shown in Figure 7. It could be observed that the addition of the MDH/SEP mixture improved the modulus, tensile strength and HDT. In the case of modulus and tensile strength, the values follow a linear trend with the final amount of FR additives. There was a decrease on the HDT values in the case of the low-load references (≈5 wt.%), due to the worsening effect of MDH. However, once a certain amount of loading was reached (≈10 wt.%), the values increased due to the enhancement effect caused by SEP. Regardless of the property, the final results of the MDH/SEP composites seemed to be in the average of the effects provided independently for both additives.

#### 3.3.2. Fire Properties

**Figure 8:**
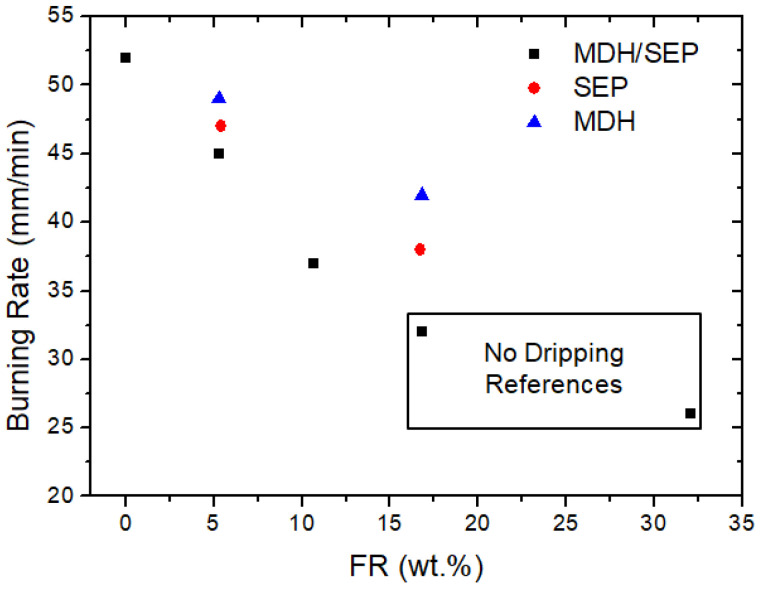
Burning rate results of the composites as a function of FR additives and their quantity.

Flame retardant virgin ABS with UL-94 HB classification is now commercially available for electrical, electronic and decorative applications [36]. Therefore, in this study, the fire performance of MDH/SEP composites was analyzed following this UL-94 HB classification, as well as the cone calorimetric tests. The results are shown in Figure 8.

Regardless of the additive, the burning rates of the materials decreased with the increasing amount of additive in the final composite. According to Figure 8, SEP provided better values than MDH when used as FR additive. However, unlike mechanical properties, the mixture of the two produced composites with better fire performance than expected from the average of both. This phenomenon was observed in the 5 to 15 wt.% range, demonstrating the synergistic effect anticipated above. In addition, Figure 8 shows that this effect is increasing with the increasing amount of additives. In this respect, the improvement for the sample loaded with 5 wt.% is about 6% compared to the expected value, while for the sample loaded with 15 wt.% it is a decrease of 25%. Furthermore, the composite containing 30 wt.% MDH/SEP had the lowest burning rate (27 mm/min versus 52 mm/min of rABS) without dripping.

Despite observing an improvement in flame retardant properties as the additive concentration increases, the upper limit of FR concentration was set at 30% because, as shown in Figure 7, for higher values the overall improvement in mechanical properties is not substantial (the Charpy impact decreases significantly).

**Figure 9:**
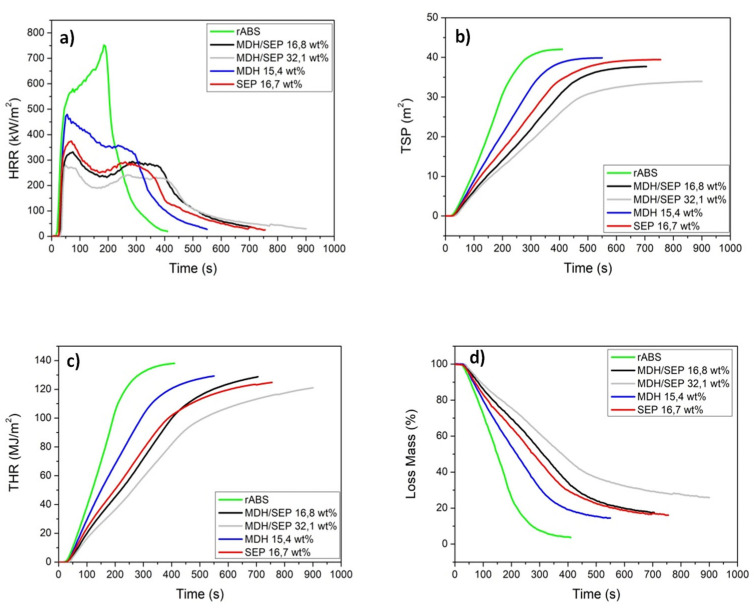
HRR (**a**), TSP (**b**), THR (**c**) and loss mass (**d**) of FR composites and rABS material.

To predict the fire behavior in real-time, cone calorimetric tests were carried out by measuring the decrease in oxygen concentration on the gases released during combustion of the samples subjected to a heat flux of 50 kW/m^2^ following the ISO 5660-1. This test provides several flammability parameters, such as time-to-ignition (TTI), heat release rate (HRR), total smoke production (TSP), total heat release (THR) and mass loss, which are shown in Figure 9 and Table 6. The TTI is an important property of the reaction to fire because it defines how fast the flaming combustion occurs when the composite is exposed to fire. Table 6 shows that the TTI increased when FR additives were incorporated into the rABS. The TTI of the mono-additivated samples showed a similar behavior, increasing by 5 s with respect to the rABS material. Furthermore, the combination of MDH and SEP increased the TTI reaching 35 s for the reference containing 15 wt.% MDH and 15 wt.% SEP.

HRR is the rate of heat generation by fire and is the most important variable in determining fire hazard [38]. The HRR curves and PHRR (the maximum peak of HRR) for FR composites are shown in Figure 9a) and Table 6, respectively. It was observed that the HRR decreased with the incorporation of FR additives in the rABS, with the largest decrease for the MDH/SEP composites. In the case of the mono-additivated materials, the HRR decreased to a greater extent than the material containing 16.7 wt.% SEP which showed a PHRR value at 374 kW/m^2^, while the material with 15.4 wt.% MDH showed a PHRR at 477 kW/m^2^. In the case of MDH/SEP composites, the HRR decreased as the amount of FR increased. The composite loaded with 32.1 wt.% MDH/SEP showed the highest reduction in HRR, reaching a PHRR at 279 kW/m^2^ compared to 752 kW/m^2^ for the rABS reference.

TSP decreased when FR additives were added in rABS (Figure 9b). The reduction on this parameter is essential, since smoke is a dangerous agent in a fire situation, mainly due to the toxicity of the CO generated. The TSP curves showed the same trend as the HRR curves, with the lowest value reached by the composite containing 32.1 wt.% MDH/SEP (that is 34 m^2^ versus 42 m^2^ for rABS, collected in Table 6).

THR analysis quantifies the heat released by combustion during a given time (Figure 9c). The results showed that the incorporation of FRs reduced the heat transfer between the rABS and the environment, with 119 MJ/m^2^ being the lowest value for the sample with 32.1 wt.% MDH/SEP.

The final flammability parameter studied was the mass loss (Figure 9d), which gives the amount of remaining polymer residue after the burning process is finished. The rABS sample showed a residue of 3.7%, indicating that most of the polymer was burnt during the process. However, the FR composites did not present that much loss, with 25.8% being the highest residue percentage for the material loaded with 32.1 wt.% MDH/SEP during the cone calorimeter test. As a conclusion of all these results, the best improvement of the fire properties of the rABS polymer was achieved with the addition of the combination of MDH and SEP (around 30 wt.% total of FR), which led to a synergy and results that were better than each FR.

## 4. Conclusions

The effect of different non-halogenated FRs on the flame retardancy and mechanical properties of a recycled acrylonitrile-butadienestyrene (rABS) material has been evaluated. Four different types of FRs were selected for this study: ATH, MDH, SEP and PAVAL. ATH, MDH and PAVAL have a flame retardancy mechanism that is based on the decomposition of the hydroxides into their corresponding oxides and water, while the SEP acts as a pure barrier against flame spread.

The first part of this study has been focused on the analysis of the thermo-mechanical and flame retardancy properties of a set of FR composites including: rABS without FR; samples containing 15 wt.% of each FR additive; and samples containing 15 wt.% of a blend of two different FR additives (7.5 wt.% of each FR additive). It was shown that from all FR additives, SEP offered the composites with the best mechanical properties, with an increase in the modulus, tensile strength and service temperature. In terms of fire performance, only the composites containing SEP passed the UL-94 HB tests. In addition, the combination of SEP with MDH resulted in composites with improved fire performance (no dripping and low burning rates). When blending two FRs, fire tests showed that only the combinations MDH + SEP (15 wt.%) and PAVAL + SEP (15 wt.%) were classified by UL-94 HB tests (no dripping and low burning rate), reaching up to 40% improvement in burning rate for the MDH + SEP sample.

The synergy observed on the blending of SEP and MDH FRs on the final composite led to the second part of this study, where the effect of different amounts of each FR on the mechanical and fire properties of the rABS material was investigated. The thermo-mechanical analysis of the MDH/SEP composites showed that the mechanical properties improved at higher SEP contents (>10%). Furthermore, the fire performance of these composites was evaluated by means of the UL-94 and the cone calorimetric tests. The best performance was achieved with the composite containing 30 wt.% FR (15 wt% of each FR). This sample was classified by the UL-94 HB test, achieving a reduction of the burning rate of 48% without dripping. In addition, this sample improved the flammability parameters such as time-to-ignition (75% increase), heat release rate (63% reduction), total smoke production (19% reduction), total heat of release (14% reduction) and residual mass after ignition (7 times increase).

As a final conclusion, this study demonstrates that the combination of SEP and MDH can be a greener alternative for the use of halogenated FRs.

## Figures and Tables

**Table 1:** First set of FR composite samples (ATH, MDH, PAVAL and SEP).

	Reference	FR 1	wt.%	FR 2	wt.%
rABS	[1]	-	-	-	-
Additives study	[2]	ATH	15	-	-
	[3]	MDH	15	-	-
	[4]	PAVAL	15	-	-
	[5]	SEP	15	-	-
Synergies study	[6]	ATH	7.5	MDH	7.5
	[7]	ATH	7.5	PAVAL	7.5
	[8]	ATH	7.5	SEP	7.5
	[9]	MDH	7.5	PAVAL	7.5
	[10]	MDH	7.5	SEP	7.5
	[11]	PAVAL	7.5	SEP	7.5

**Table 2:** Results of the characterization of the FR additives.

Flame Retardant	Particle Size (µm)	Density (g/cm^3^)	Water Release (%)	Temperature at Which Water is Realesed (°C)
ATH	20.5 ± 17.0	2.5	31	293
MDH	16.1 ± 9.6	2.4	27	400
PAVAL	1.9 ± 0.9	1.62	13	275
SEP	<5 (aggregates)	1.04	10	100

**Table 3:** Thermo-mechanical properties of the different FR composites additivated with ≈15 wt.%.

	Reference	FR	Total FR (wt%) *	Modulus (MPa)	σ (MPa)	ε (%)	Charpy (kJ/m^2^)	HDT (°C)
rABS	[1]	-	0	2435 ± 23	40 ± 1	20 ± 5	6.0 ± 0.2	80 ± 1
Flame retardant study	[2]	ATH	16.6	3044 ± 18	35 ± 1	14 ± 4	2.6 ± 0.2	77 ± 1
	[3]	MDH	15.1	3118 ± 120	39 ± 1	10 ± 3	2.7 ± 0.2	76 ± 1
	[4]	PAVAL	16.5	2998 ± 37	42 ± 1	7 ± 2	2.1 ± 0.2	77 ± 1
	[5]	SEP	16.7	3894 ± 50	51 ± 1	3 ± 1	1.8 ± 0.2	89 ± 1
Synergy study	[6]	ATH + MDH	16.6	2958 ± 46	36 ± 1	11 ± 4	2.8 ± 0.3	79 ± 1
	[7]	ATH + PAVAL	16.5	2996 ± 41	39 ± 2	6 ± 1	2.5 ± 0.2	76 ± 3
	[8]	ATH + SEP	16.8	3563 ± 25	45 ± 1	6 ± 1	2.0 ± 0.3	85 ± 1
	[9]	MDH + PAVAL	16.5	3061 ± 94	40 ± 1	8 ± 1	2.4 ± 0.1	79 ± 1
	[10]	MDH + SEP	16.8	3669 ± 36	46 ± 1	6 ± 1	2.1 ± 0.2	84 ± 1
	[11]	PAVAL + SEP	16.1	3440 ± 19	45 ± 1	4 ± 1	2.1 ± 0.2	82 ± 1

* The residual values were corrected by subtracting the residual value obtained in the pure rABS.

**Table 4:** Results of the burning test for FR composites.

	Reference	FR	wt%	Dripping	Linear Burning Rate (mm/min)	Flammability Rating ^a^
rABS	[1]	-	0	YES	52	No rating
Flame retardant study	[2]	ATH	16.6	YES	41	No rating
	[3]	MDH	15.1	YES	42	No rating
	[4]	PAVAL	16.5	YES	42	No rating
	[5]	SEP	16.7	YES	38	HB
Synergy study	[6]	ATH + MDH	16.6	YES	43	No rating
	[7]	ATH + PAVAL	16.5	YES	42	No rating
	[8]	ATH + SEP	16.8	NO	41	No rating
	[9]	MDH + PAVAL	16.5	YES	42	No rating
	[10]	MDH + SEP	16.8	NO	32	HB
	[11]	PAVAL + SEP	16.1	NO	38	HB

^a^ HB Horizontal Burn Rate: Slow horizontal burning on a 3 mm thick specimen with a burning rate of less than 75 mm/min.

**Table 5:** List of the samples prepared to evaluate the influence of FR in the final composites.

	Reference	FR 1	wt%	FR 2	wt%	Total FR wt% (TGA)
rABS	[1]	-	-	-	-	-
SEP	[2]	SEP	5	-	-	5.4
	[3]	SEP	15	-	-	16.7
MDH	[4]	-	-	MDH	5	4.9
	[5]	-	-	MDH	15	15.4
MDH/SEP	[6]	SEP	2.5	MDH	2.5	5.3
	[7]	SEP	5	MDH	5	10.7
	[8]	SEP	7.5	MDH	7.5	16.8
	[9]	SEP	15	MDH	15	32.1

**Table 6:** Data of Composites at 50 kW/m^2^ from cone calorimeter tests.

	Reference	FR 1	wt%	FR 2	wt%	Total FR wt% (TGA)	TTI (s)	HRR (kW/m^2^)	TSP (m^2^)	THR (MJ/m^2^)	Residue (%)
rABS	[1]	-	-	-	-	-	20	752 ± 50	42 ± 1	138 ± 1	3.7 ± 0.2
SEP	[3]	SEP	15	-	-	16.7	25	374 ± 5	39 ± 1	124 ± 2	16.1 ± 0.3
MDH	[5]	-	-	MDH	15	15.4	25	477 ± 30	42 ± 1	128 ± 2	18.8 ± 0.2
MDH/SEP	[8]	SEP	7.5	MDH	7.5	16.8	30	337 ± 2	38 ± 4	130 ± 1	17.6 ± 0.1
	[9]	SEP	15	MDH	15	32.1	35	279 ± 6	34 ± 2	119 ± 1	25.8 ± 0.3

## Data Availability

Not applicable.
